# Visual inspection with acetic acid (VIA) positivity among female sex workers: a cross-sectional study highlighting one-year experiences in early detection of pre-cancerous and cancerous cervical lesions in Kampala, Uganda

**DOI:** 10.1186/s13027-021-00373-4

**Published:** 2021-05-11

**Authors:** Gertrude Namale, Yunia Mayanja, Onesmus Kamacooko, Daniel Bagiire, Agnes Ssali, Janet Seeley, Robert Newton, Anatoli Kamali

**Affiliations:** 1 MRC/UVRI and LSHTM Uganda Research Unit, P.O Box 49, Entebbe, Uganda; 2 London School of Hygiene &Tropical Medicine, London, United Kingdom; 3 University of York, York, United Kingdom

**Keywords:** Female sex workers (FSWs), visual inspection with acetic acid (VIA), cervical intra-epithelial neoplasia (CIN), screening, HIV

## Abstract

**Background:**

Although cervical cancer is preventable, most women in sub-Saharan Africa (SSA) do not receive routine screening and few treatment options exist. Female Sex Workers (FSWs) are among the Ugandan female population at highest risk of acquiring sexually transmitted infections (STIs) including HIV and human papilloma viruses (HPV), the cause of cervical cancer. We report one-year experiences of visual inspection with acetic acid (VIA) positivity among FSWs in the early detection of pre-cancerous and cancerous cervical lesions in Kampala, Uganda.

**Methods:**

Between June 2014 and July 2015, we enrolled FSWs into a cross-sectional study at a research clinic. The women were screened using the VIA method (application of 3–5 % acetic acid to the cervix). All VIA positive women were referred to a tertiary hospital for colposcopy, biopsy, and immediate treatment (if indicated) at the same visit according to national guidelines. Data on socio-demographic, sexual behaviour, sexual reproductive health and clinical characteristics were collected. We used logistic regression to identify factors associated with VIA positivity.

**Results:**

Of 842 women assessed for eligibility, 719 (85 %) of median age 30 (IQR 26, 35) were screened, and 40 (6 %) women were VIA positive. Of the 24 histology specimens analysed, 6 showed inflammation, only 1 showed cervical intraepithelial neoplasia (CIN) 1, 13 women showed CIN2/3, while 4 women already had invasive cervical cancer. The overall prevalence of HIV was 43 %, of whom only 35 % were receiving ART. In the age-adjusted analysis, VIA positivity was more likely among women who reported having > 100 life-time partners (aOR = 3.34, 95 %CI: 1.38–8.12), and HIV positive women (aOR = 4.55; 95 %CI: 2.12–9.84).

**Conclusions:**

We found a relatively low proportion of VIA positivity in this population. The experience from our program implies that the VIA results are poorly reproducible even among a category of trained professional health workers. VIA positivity was more likely among women with a high number of sexual partners and HIV infection. Interventions for improving cervical cancer screening should be recommended as part of HIV care for FSWs to reduce the disease burden in this population.

## Background

Cervical cancer continues to be a major public health problem worldwide [1]. Globally, cervical cancer is the fourth most common cancer among women, with more than 500,000 new cases occurring annually [2, 3]. More than 85 % of the women who die of cervical cancer annually live in low- and middle-income countries (LMICs) with sub-Saharan Africa (SSA) carrying the highest burden[4]. In Uganda, studies have shown an overall trend of elevated cervical cancer incidence, with over 80 % of women diagnosed with advanced disease [5, 6]. Cervical cancer is a preventable disease if precancerous lesions are detected early through effective screening programs. World Health Organization (WHO) recommends visual inspection with acetic acid (VIA) as the primary approach for cervical screening in resource-constrained settings [2]. In principle, this screening method is a less complex technique and can be performed by trained healthcare providers with different backgrounds e.g. doctors, and nurses [7].

Compared to women in the general population, female sex workers (FSWs) are among the Ugandan female populations at the highest risk of acquiring sexually transmitted infections (STIs) including HIV and human papilloma viruses (HPV), the cause of cervical cancer [8]. Earlier reports show that high risk sexual behaviour and HIV infection are associated with an increased risk of HPV infection among FSWs [9]. Published data also indicates that Cervical Intraepithelial Neoplasia (CIN), and invasive cervical cancer are more common in high-risk women compared to their counterparts [10]. WHO recommends that high-risk women including FSWs should be screened more often than other women in the general population [2]. However, due to a range of social-cultural challenges including stigma and discrimination, FSWs are usually hard to reach and do not receive regular cervical screening services [11].

Although VIA is the standard of care in many LMICs, the diagnostic accuracy in detecting precancerous and invasive cancerous cervical lesions varies across studies [12]. For example in a meta-analysis on the accuracy of VIA in LMICs, healthcare providers correctly identified between 41 and 92 % of women with cervical intraepithelial neoplasia (CIN) grade 2 or worse [13]. The reference standard test (or gold standard) that was selected for this review was either a combination of colposcopy and histology, or histology without colposcopy [13]. These data suggest that the VIA method has some limitations. According to previous authors, the important causes of variations could be due to highly subjective results dependent on individual interpretation, quality assurance and training, the variation of light source, the procedure for 3–5 % acetic acid preparation, and its storage [14]. Thus, acquiring good VIA skills is mandatory to lower the impact of these limitations.

However, the health providers’ VIA screening skills have been shown to improve significantly over time with continuous exposure and practice [15]. For example in Ghana, the health care providers who had been trained up to seven years with regular supervision maintained high visual inspection performance [16]. In Uganda, the Ministry of Health (MoH) Non-Communicable Diseases (NCDs) division has developed comprehensive national cervical cancer prevention and control guidelines [17] to strengthen the capacity of health-care providers in their health care settings. Continuous experience, availability of standardized protocols, quality checks, and routine supervision are crucial for improving VIA accuracy.

Although scaling up of VIA-based programs into national programs is already taking place in many SSA countries [7], data on the evaluation of service delivery among high-risk women including FSWs is still scarce. Understanding the implementation of VIA screening programs among FSWs is an essential step in developing effective interventions for the prevention and control of cervical cancer in this population [18, 19].

## Methods

### Aim, design and setting

We conducted a cross-sectional study between June 2014 and July 2015 to investigate VIA positivity among FSWs in the early detection of pre-cancerous and cancerous cervical lesions at the Good Health for Women Project (GHWP) clinic in Kampala, Uganda. The clinic was established in 2008 to study HIV and STIs epidemiology and to implement HIV/STIs prevention among FSWs. The clinic, a stand-alone clinic located in a peri-urban suburb in Southern Kampala, the capital city of Uganda reported a high HIV/STIs prevalence combined with high-risk behaviour in 2011 [8]. The clinic offers HIV care and preventative services, sexual and reproductive health (SRH) services such as syndromic management of STIs, family planning, antenatal care, promotion and distribution of free condoms, and risk reduction counselling to both HIV positive and negative FSWs.

### Participants

The participants were asymptomatic, healthy and previously unscreened FSWs who were receiving targeted HIV prevention, care, and treatment services at a dedicated GHWP clinic. FSWs were defined as women having sex with men in exchange for money, favours or other goods either regularly or casually at least once in the past 12 months.

### Eligibility criteria

The eligibility criteria for participation in the study included: (1) being a FSW aged ≥ 14 years old; (2) a resident of the catchment area; (3) no previous history of total hysterectomy; (4) not using any intra-vaginal medication at the time of the study visit; (5) ability to provide informed consent; (6) had not been previously diagnosed with cervical cancer.

We excluded those; (1) who were very ill requiring emergency care; (2) women with evidence of acute STIs; (3) women less than 12 weeks after delivery; (4) pregnant women; (5) currently menstruating; and (6) women with known allergy to acetic acid. For women who were menstruating, the screening procedure was deferred until menstruation was over.

### VIA program and procedures

#### a) VIA training and quality assurance

Before implementation of the VIA program, the nurses and clinicians were given a 5-days training using the WHO guidelines [20]. The training included a mixture of interactive and practical sessions and discussions. The facilitators included a senior gynaecologist and midwife from St Francis hospital Nsambya, a tertiary referral hospital. Following hands-on experience under supervision for two consecutive weeks, the trained staff were allowed to screen the women independently. Periodic reorientation sessions and routine quality checks were conducted to improve quality assurance. The VIA screening protocol was distributed in the clinic to use during the screening of the women. Awareness campaigns among the FSWs were performed by the trained staff during the health education activities at the GHWP clinic. Information, education and communication (IEC) materials were used to assist in this process.

#### b) Recruitment and VIA screening procedure

The women were recruited during their three-monthly routine clinic visits. Those who were interested and eligible for screening were offered the screening test after obtaining informed consent. VIA was performed according to the Uganda MoH guidelines [17]. The women underwent a routine vaginal examination using a sterile bivalve self-retaining vaginal speculum. The squamo-columnar junction (SCJ) was identified and any secretions or exudate were cleaned off before applying a cotton swab soaked in a 3–5 % freshly prepared acetic acid solution to the cervix. The results were recorded after 1–3 min using a bright halogen lamp. The VIA result was classified as negative, positive or inconclusive. Pre-cancerous and cancerous lesions were recorded positive when a well-defined, dense aceto-white area with regular margins appeared attached to the SCJ or if the whole cervix or cervical growth turned white. The test was reported negative if no change was observed or suspicious for invasive cancer, i.e., if no growth or ulcerative lesion was observed [21]. If there was any uncertainty about the lesion observed, such women were recalled for rescreening. All VIA positive women were immediately informed and offered accompanied referral to the senior gynaecologist at St Francis hospital Nsambya for colposcopy, and further management. The participants had a pregnancy test followed by a general physical and gynaecological exam. The entire screening procedure and procedures done at referral facility were paid for by project to ensure that all women who tested positive receive treatment.

#### c) Clinical management procedure

The women with a positive VIA result were referred to a collaborating tertiary hospital on the same day for further evaluation in accordance with the “screen and treat” program [17]. At the referral facility, colposcopy was done to grade the lesions and biopsies taken from areas which looked to be suspicious or abnormal. Colposcopic findings were reported as normal, inflammation, probable low- or high-grade precancerous lesions, or suspected invasive cancer. The colposcopic findings and procedure were explained to the woman. Immediate treatment with cryotherapy was offered in the same session, if the lesion involved < 75 % of the transformational zone, did not involve the endocervix or if there was no evidence of invasive cancer. Women with lesions involving > 75 % of the transformation zone, endocervical involvement or suspicious of invasive cancer were offered loop electrosurgical excision procedure (LEEP) to remove abnormal cervical tissues [22]. Similarly, those who were found to have invasive cervical cancer were managed as per the national guidelines [17]. All the women who were treated were given a course of antibiotics for a week. Cervical biopsy specimens were analyzed and the results were reported using the CIN system [23] and explained to the participant during the follow-up clinic visit 1–4 weeks later.

If any STIs were diagnosed, the women were offered free treatment using a syndromic approach at the GHWP clinic. The women who tested HIV positive were enrolled into care. Pregnant women were provided with iron and folate supplements according to Uganda national clinical guidelines [24]. The women were also encouraged to invite their male regular partners (MRPs) to the clinic for free HIV/STIs screening and treatment as well as counselling on safe medical male circumcision and condom use. The details of all the medical procedures remained totally confidential.

### Laboratory procedures

As part of routine clinical care, blood samples were collected by an experienced and trained health worker for assessing HIV sero-positivity at the GHWP clinic laboratory.Participants received confidential HIV pre-test and post-test counselling on the study visit. HIV status was determined by performing a rapid HIV test (Determine, Statpak, Unigold) on a serum sample according to the Uganda National algorithms [25] for HIV testing HIV status was recorded as negative or positive. The HIV test results were linked to participants by their unique study identification number. Samples of urine were taken off for routine testing for the presence of Human Chorionic Gonadotropic (hCG) at the GHWP clinic laboratory. The biopsy cervical specimens were analysed at the referral hospital laboratory. The 3–5 % acetic acid was freshly prepared at the GHWP clinic laboratory by carefully adding 5 mL of glacial acetic acid into 95 mL of distilled water and mixing thoroughly. Unused acetic acid was discarded at the end of the day.

### Data collection and study measures

After obtaining written informed consent, eligible FSWs were consecutively enrolled and face-to-face semi-structured interviews conducted. Counsellors collected data on socio-demographic characteristics, sexual behaviour and clinical characteristics. The primary outcome of this study was VIA positivity which was categorised as negative or positive. Socio-demographic measures included age, marital status, education level, alcohol use, and illicit drug use. Alcohol use was assessed by using a standardized WHO Alcohol Use Disorders Identification Test (AUDIT) [26]. Alcohol use was classified into three categories i.e. harmless or low-risk drinkers: score 1–7, harmful or high-risk drinkers: score 8–19 and alcohol dependent: score 20+. The sexual behaviour characteristics included: the total number of lifetime partners, condom use at last sexual intercourse, and age at first sexual intercourse. Sexual and reproductive health characteristics included: age at first pregnancy, parity, family planning use in the last 12 months, and family planning method. Participants’ clinical characteristics included; the presence of STI symptoms in the last 12 months, HIV status, receiving ART, VIA result, and Biopsy result. Presence of STI symptoms was determined if a FSW self-reported having had any symptoms suggesting STIs in the last 12 months including vaginal discharge, genital ulcer, and pain. HIV status was categorised as negative or positive. The histopathological findings were categorized into five categories: normal, inflammation, CIN 1 (low-grade cervical lesions), CIN 2/3 (high-grade cervical lesions), and invasive cervical cancer.

### Statistical analysis

Data were double entered in Microsoft Access, cleaned, and exported to STATA 14.0 (StataCorp, College Station, TX, USA) for analysis. We resolved discrepancies by checking the source documents for clarification. Categorical demographic and clinical characteristics were summarized by counts and percentages. Continuous variables were summarized by means and standard deviations or medians and interquartile ranges. The proportion of those who were VIA positive was analysed by the different demographic, sexual behavioural and clinical characteristics. Factors for which the association attained statistical significance on log likelihood ratio test (LRT) of *p* < 0.20 were selected for the multivariable logistic regression model. Logistic regression models were fitted to identify factors associated with VIA positivity at unadjusted analysis. Factors were retained in the final multivariable logistics regression model if their inclusion did not make the fit of the model significantly worse at the 5 % level on a likelihood ratio test (LRT).

## Results

### Recruitment profile

**Fig. 1:**
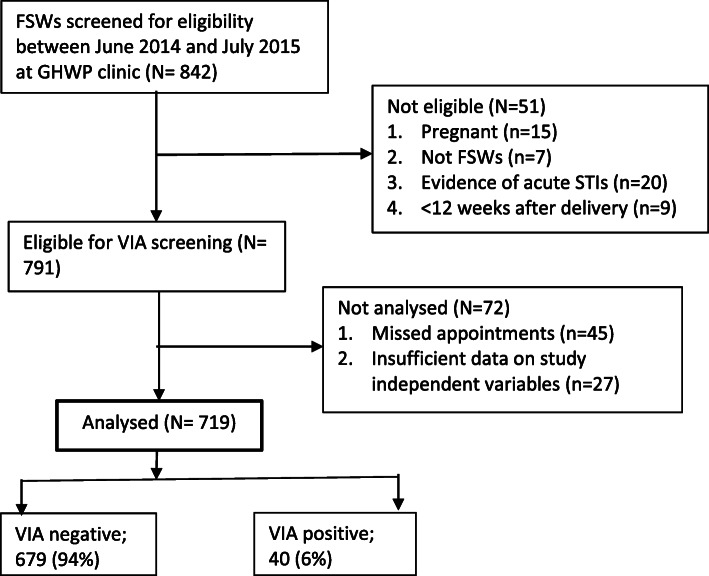
Recruitment profile of FSWs for VIA screening at Good Health for Women Project (GHWP) clinic in Kampala, Uganda (2014–2015)

During the study period, 842 women were screened to participate at GHWP clinic (Fig. 1), of these, 123 (15 %) were excluded: 51 (6 %) were ineligible; (pregnant [*n* = 15], not FSWs [*n* = 7], evidence of acute STIs [*n* = 20], and < 12 weeks after delivery [*n* = 9]); 72 (9 %) were not analysed; (missed appointments [*n* = 45], and insufficient data on study independent variables [*n* = 27]). Thus, a total of 719 FSWs were included in the analysis (Fig. 1).

### Participants’ characteristics

Seven hundred and nineteen women were included in the analysis. Their median age was 30 years (IQR, 26–35). More than half (54 %) of the FSWs were aged 25–34 years, more than half (56 %) attained at least primary education and above, and about two thirds (64 %) were divorced/separated. About a third had ever smoked, and 12 % reported being alcohol dependent. 43 % reported to have had at least 50 lifetime sexual partners, and about half (52 %) reported using condoms during the last sexual intercourse.

More than half (55 %) reported their first sexual intercourse between the age of 15–19 years and the median age 16 years (IQR, 15–18). Regarding age at first pregnancy, the median was 17 years (IQR, 16–19) and more than half (55 %) had their first pregnancy at < 18 years. More than 40 % had 1–2 children, nearly two thirds (58 %) were using family planning in the past 12 months, with the injectable contraceptive being the most preferred method (25 %). Most (71 %) reported not having signs and symptoms of STIs in the last three months. HIV prevalence was 43.8 %, out of whom only 35 % were receiving ART. Overall VIA positivity was 6 % (40/719). Of the 24 histology cervical specimens analysed, 6 women showed normal or inflammation, only 1 showed CIN1, about half [13] of the women showed CIN2/3, while 4 women already had invasive cervical cancer. (Table 1).

**Table 1:** Characteristics of FSWs who were screened for pre-cancerous and cancerous lesions using VIA at Good Health for Women Project (GHWP) clinic in Kampala, Uganda (2014–2015

Socio-demographic characteristics		
**Characteristic**	**Frequency**	**Percentage (col % )**
**Age, years **(***N* = 717)**		
Median, 30 (IQR, 26–35)		
18–24	138	19
25–34	386	54
≥ 35	193	27
**Education level ((*N* = 687)**		
Less than primary	303	44
Primary or greater	384	56
**Current marital status (*N* = 713)**		
Married/cohabiting	109	15
Widowed	40	6
Separated/divorced	458	64
Never married	106	15
**Smoker (*N* = 719)**		
Never	501	70
Ever	218	30
**Alcohol use (*N* = 651)**		
Low risk	320	49
Harmful/high risk	251	39
Alcohol dependent	80	12
**Sexual behaviour characteristics**		
** Total number of lifetime partners (*N* = 707)**		
≤ 50	305	43
51–100	268	38
> 100 or cannot remember	134	19
**Condom use at the last sexual intercourse (*N* = 634)**		
No	306	48
Yes	328	52
**Age at first sexual intercourse, years (*N* = 712)**		
Median; 16 (IQR: 15, 18)		
< 15	266	37
15–19	391	55
> 20	55	8
**Sexual and reproductive health characteristics**		
** Age at first pregnancy, years (*N* = 671)**		
Median; 17 ( IQR, 16–19)		
< 18	369	55
18+	302	45
**Parity (*N* = 719)**		
Nulliparous	47	7
1–2	311	43
3–4	254	35
5+	107	15
**Family planning use in the last 12 months (*N* = 719)**		
Yes	420	58
No	299	42
**Family planning method (*N* = 719)**		
None	299	42
Condoms	139	19
Injectable contraceptive	176	25
Oral contraceptive	45	6
Implants (progesterone)	23	3
Other method	37	5
**Participants’ clinical characteristics**		
**Presence of signs and symptoms of STIs in the last three months (*N* = 719)**		
No	512	71
Yes	207	29
**HIV status (*N* = 719)**		
Positive	315	44
Negative	404	56
**Receiving ART (*N* = 719)**		
Yes	252	35
No	467	65
**VIA result (N = 719)**		
Negative	679	94
Positive	40	6
**Biopsy result (*N* = 24)**		
Normal or Inflammation	6	31
Low grade lesions (CIN I)	1	4
High grade lesions (CIN II-III)	13	50
Invasive cancer	4	15

### Colposcopic findings and management of VIA positive FSWs at the referral hospital

**Fig. 2:**
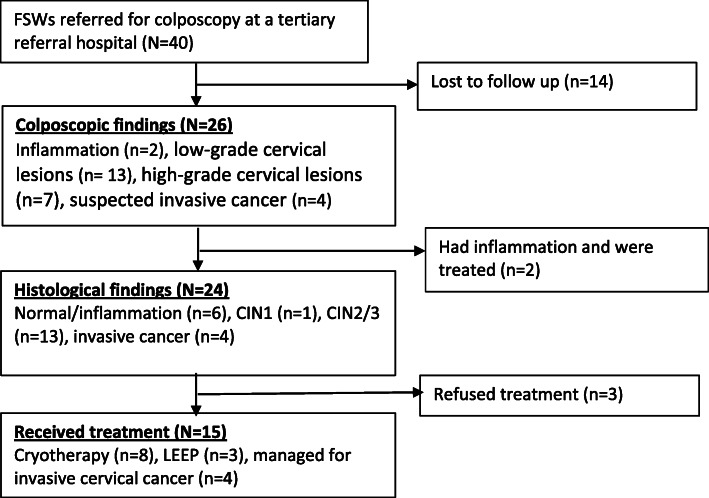
The flow of the referred VIA positive FSWs at the referral tertiary hospital in Kampala, Uganda (2014–2015)

From the 40 VIA positive women, 26 (65 %) were referred for colposcopy and 14 (35 %) were lost to follow up (LTFUP). Of the 26 colposcopic assessments done, 2 women had inflammation, 13 had low-grade precancerous lesions, 7 had high-grade lesions, while 4 had suspected invasive cancer. Of the 14 women who had CIN2/3 & CIN 1 from the biopsy results, 11 accepted immediate treatment (cryotherapy, 8; LEEP,3). The 4 women with invasive cervical cancer also complied with the treatment. The remaining 3 refused treatment. (Fig. 2).

### VIA positivity and associated factors

At unadjusted analysis, VIA positivity more likely among HIV positive women (uOR = 4.42; 95 %CI: 2.13–9.14). In age-adjusted analysis, VIA positivity was less likely among women aged 14–24 years old (uOR = 0.18; 95 %CI: 0.04–0.81), and was more likely women who reported having > 100 lifetime partners (aOR = 3.34, 95 %CI: 1.38–8.12), and HIV positive women (aOR = 4.55; 95 %CI: 2.12–9.84). (Table 2).

**Table 2:** Characteristics of FSWs and association with VIA positivity in Kampala, Uganda

Characteristic	N	# VIA positive (col%)	Unadjusted OR (95 %CI)	Age-adjusted OR (95 %CI)	Fully adjusted OR (95 %CI)
**Current marital status (*N* = 713)**					
Married/cohabiting	109	3 (2.8)	0.73 (0.17–3.18)	0.64 (0.15–2.75)	0.52 (0.10–2.58)
Widowed	40	4 (10.0)	2.65 (0.70–10.09)	2.20 (0.58–8.24)	1.74 (0.39–7.77)
Separated/divorced	458	29 (6.3)	1.67 (0.60–4.67)	1.33 (0.48–3.70)	2.25 (0.36–4.06)
Single	106	4 (3.8)	1.0	1.0	1.0
**Smoker (*N* = 719)**					
Never	501	26 (5.2)	1.0	1.0	1.0
Ever	218	14 (6.4)	1.20 (0.66–2.32)	1.21 (0.65–2.27)	1.12 (0.59–2.14)
**Alcohol use pattern following AUDIT score (*N* = 651)**					
Harmless or low-risk drinkers	320	21 (6.6)	1.31 (0.46–3.72)	1.11 (0.40–3.11)	1.21 (0.42–3.50)
Harmful or high-risk drinkers	251	14 (5.6)	1.11 (0.38–3.30)	1.04 (0.36–3.03)	1.11 (0.38–3.30)
Alcohol-dependent	80	4 (5.0)	1.0	1.0	1.0
**Number of lifetime partners (*N* = 707)**					
≤ 50	305	15 (4.9)	1.0	1.0	1.0
51–100	268	10 (3.7)	0.75 (0.35–1.66)	0.74 (0.34–1.64)	1.07 (0.40–2.86)
> 100	134	12 (9.0)	1.82 (0.88–3.78)	1.69 (0.81–3.52)	**3.34 (1.38–8.12)**
**Number sexual partners in last month (*N*= 594)**					
< 5	234	14 (6.0)	1.0	1.0	1.0
5–19	137	6 (4.4)	0.73 (0.29–1.86)	0.82 (0.32–2.07)	0.63 (0.23–1.64)
20–49	88	3 (3.4)	0.57 (0.17–1.94)	0.67 (0.20–2.29)	0.53 (0.16–1.84)
50+	135	7 (5.2)	0.87 (0.36–2.09)	0.98 (0.41–2.36)	0.58 (0.23–1.54)
**Condom use in the last 3 months (*N* = 634)**					
Inconsistent use	306	21 (6.9)	1.0	1.0	1.0
Consistent use	328	17 (5.2)	0.75 (0.41–1.40)	0.80 (0.43–1.48)	1.05 (0 0.51–2.17)
**Age at first sex (*N* = 712)**					
< 15	266	16 (6.0)	1.0	1.0	1.0
15–19	391	22 (5.6)	0.94 (0.50–1.75)	0.94 (0.50–1.74)	0.89 (0 0.45 -1.78)
> 20	55	1 (1.8)	0.30 (0.04–2.23)	0.36 (0.04–2.64)	0.30 (0 0.04 -2.29)
**Age at first pregnancy (*N *= 671)**					
< 18	369	17 (4.6)	1.0	1.0	1.0
18+	302	20 (6.6)	1.44 (0.77–2.69)	1.49 (0.80–2.77)	1.49 (0 0.76–2.91)
**HIV status (*N *= 719)**					
Negative	404	31 (7.7)	1.0	1.0	1.0
Positive	315	9 (2.9)	**4.42 (2.13–9.14)**	**3.90 (1.37–11.09)**	**4.55 (2.12–9.84)**
**Signs and symptoms of STIs (*N* = 719)**					
No	512	28 (5.5)	1.0	1.0	1,0
Yes	207	12 (5.8)	1.06 (0.55–2.04)	1.00 (0.51–1.97)	1.06 (0.55–2.02)

## Discussion

This study reports one-year experiences of VIA-based cervical screening among asymptomatic, apparently healthy and previously unscreened Ugandan FSWs. Our experience has shown that the proportion of VIA positivity was surprisingly low in this high-risk population. Similar discrepancies were also reported in the Nigerian and Malawian studies [27, 28]. In contrast, a higher prevalence of VIA positivity was observed among South African FSWs [18]. According to previous reports, these variations could be due to the health provider’s skills and experience in the interpretation of the VIA result [14]. In this study, the most likely explanation is that, although our midwives and clinicians were trained, it’s possible that not all of them attained the same level of skill. Also, the VIA test was a new service at the GHWP clinic and our health workers did not have previous experience. We believe that perhaps with more practice and experience, their visual inspection accuracy would improve. This study provides a possible example of difficulties in interpreting VIA results even when VIA is used in the best possible circumstances.

Additionally, the low VIA positivity in this study could also have arisen from the fact that our nurses were more likely to label unclear visual inspection findings as negative, in order not to miss cases of inflammation and STIs given that these were FSWs at high risk of infection. Thus, lesions classified as inflammation may have been low-grade precancerous lesions, resulting in higher false-negative rates and a subsequent low VIA positivity rate. Although different study populations have reported high proportions of VIA positivity [18, 29], the findings in the present study reveal that cervical cancer is still a significant public health problem in this population. This calls for routine screening service provision given that early detection and treatment is more cost-effective than treating advanced disease [30].

Interestingly, in this study, a high proportion of VIA positive women were considered positive after a colposcopic examination. The rate of colposcopic positivity in this study was higher than what was reported in Côte d’Ivoire [31]. This high achievement in performance is relevant for scaling up cervical cancer screening and treatment programmes using VIA even at lower-level facilities. Hence, our results indicate that visual screening tests are promising methods for the early detection of pre-cancerous and cervical cancerous lesions in key populations. The apparent subjectivity of VIA as a method can be overcome with the high-quality, competency-based training and quality assurance approach used in many studies [32, 33].

We also report a high burden of cervical dysplasia among these previously unscreened high-risk women. Our histology results show that almost half of the referred women had CIN2/3. The CIN2/3 histology results in our study were higher than those reported earlier [29]. It is possible that a large percentage of FSWs were infected with high-risk HPV. FSWs constitute a very high-risk group for HIV as well as HPV infections [34]. Across SSA, the pre-cancerous and invasive cervical cancer disease burden among high-risk women is substantial [19, 34]. However, even though our FSWs regularly received vaginal examinations at GHWP clinic, the data gathered in this study calls for the need to perform regular cervical cancer screening in this population.

With regard to factors associated with VIA positivity, our results show that it was more likely among HIV positive women and those with multiple sexual partners. This is consistent with earlier epidemiological reports among FSWs [19, 35]. However, this is not particularly surprising because HIV is a known risk factor for increased VIA positivity and is common among FSWs worldwide [19, 29]. It may be argued that, since HIV and HPV are both STIs, sharing common modes of transmission, women engaging in riskier sexual practices and behaviours may have a greater likelihood of having HIV and/or STIs infected partners leading to greater exposure to both viruses. In countries with high HIV prevalence like Uganda [36], the integration of cervical cancer screening into routine HIV care provides an ideal platform to reach HIV positive women.

### Implications for policy and clinical practice

These results have important implications for efficient service delivery of cervical screening programs among FSWs. The VIA test was mostly performed by trained nurses who are the main providers of these services throughout SSA. However, given the heterogeneity of results across studies and the relatively low rates in ours, we are clearly underestimating the true burden of disease. This means that VIA, in our hands had low sensitivity. More broadly, it is not a very reproducible test. That said, we found some (but certainly not all) cases and linked them effectively into care. This same day linkage of the VIA screen-positives to the diagnosis and treatment facility was critical for the successful implementation of our program. Our study, therefore, provides important information about the feasibility of the “screen and treat” strategy for preventing cervical cancer, which has been recommended by the WHO for low-resource countries [2]. In Uganda, the “screen and treat” strategy has been advocated [17, 37], but these services may not be available in most facilities especially at primary health care level. In the context of FSWs, it is even more important to screen and treat on the same day given that this is a highly mobile population [38, 39]. Cervical cancer screening and treatment of CIN using “screen and treat” strategy needs to be integrated into targeted interventions that are ongoing for HIV prevention, care and support for FSWs. Based on our findings, there is a need to have networks and collaborations among FSW-led organisations to set up acceptable and accessible health services that integrate cervical cancer screening and HIV/STIs care.

Our study had some strengths and limitations. First, this being our first VIA screening experience we did not have a systematic reference test to confirm whether VIA positive or negative tests were correct. However, importantly, VIA remains highly related to the health worker skills, subjective nature of decision and is subject to misclassification bias, leading to possible under or over identification of cervical lesions [12]. Secondly, because we used a syndromic approach to manage STIs in our clinic due to financial limitations, there was no systematic detection of STIs as co-factors/confounders in the laboratory. Additionally, we did not investigate the prevalence of HPV and its subtypes to correlate HPV and VIA also due to financial limitations. Thirdly, for all the women who were referred for a colposcopic procedure, some were lost to follow up. Therefore, the information on VIA positive women that were referred for colposcopy is not sufficient to adequately determine the real picture of cervical lesions in this population. Importantly, a high proportion of VIA positive women were considered positive after a colposcopic examination. This high rate of CIN2/3 detection shows that VIA had reasonably high HPV detection rates. Lastly, since this was a single-site study, the results might not be generalizable to other key population clinics in Kampala. However, despite all these challenges, we believe that our experiences presented here can be useful to other key population settings in the country. The results achieved and lessons learned can serve as a guide for other low-resource countries. Also, the findings in this study add to the existing knowledge of cervical cancer screening interventions among key populations in sub-Saharan Africa.

## Conclusions

The proportion of VIA positivity that we identified was comparatively low in this population compared with studies from elsewhere in sub-Saharan Africa. The experience from our program implies that the VIA results are poorly reproducible even among a category of trained professional health workers. Our findings also suggest the need for systems to ensure quality assurance which is critical for health provider performance on cervical screening. VIA positivity was associated with a high number of sexual partners and HIV infection. Interventions aimed at improving cervical cancer screening should be recommended as part of HIV care for FSWs to reduce the preventable cervical cancer burden and associated mortality in this population.

## Data Availability

The data used to support the findings of this study are available at MRC/UVRI and LSHTM Uganda Research Unit, and are available from the corresponding author upon reasonable request and with permission from MRC/UVRI and LSHTM Uganda Research Unit.

## Abbreviations

AIDS: Acquired Immunodeficiency Syndrome
CIN: Cervical intra-epithelial neoplasia
FSWs: Female sex workers
GHWP: Good Health for Women Project
HIV: Human Immunodeficiency Virus
IQR: Interquartile range
LMICs: Low and middle income countries
LSHTM: London School of Hygiene and Tropical Medicine
MRC: Medical Research Council
MRPs: Male regular partners
OR: Odds ratio
SD: Standard deviation
SSA: Sub-Saharan Africa
UNCST: Uganda National Council of Science Technology
UVRI: Uganda Virus Research Institute
VIA: Visual inspection with acetic acid
WHO: World Health Organisation

## References

[1] Pimple S, Mishra G, Shastri S (2016). Global strategies for cervical cancer prevention. Curr Opin Obstet Gynecol.

[2] Organization WH. Comprehensive cervical cancer prevention and control: a healthier future for girls and women. Geneva, Switzerland: World Health Organization Press; 2013. 2014.

[3] Arbyn M, Castellsagué X, de Sanjosé S, Bruni L, Saraiya M, Bray F (2011). Worldwide burden of cervical cancer in 2008. Annals of oncology.

[4] Ferlay J, Shin HR, Bray F, Forman D, Mathers C, Parkin DM (2010). Estimates of worldwide burden of cancer in 2008: GLOBOCAN 2008. International journal of cancer.

[5] Odida M, Sandin S, Mirembe F, Kleter B, Quint W, Weiderpass E (2011). HPV types, HIV and invasive cervical carcinoma risk in Kampala, Uganda: a case-control study. Infectious Agents Cancer.

[6] Wabinga HR, Nambooze S, Amulen PM, Okello C, Mbus L, Parkin DM (2014). Trends in the incidence of cancer in Kampala, Uganda 1991–2010. International journal of cancer.

[7] Coleman JS, Cespedes MS, Cu-Uvin S, Kosgei RJ, Maloba M, Anderson J (2016). An insight into cervical cancer screening and treatment capacity in sub-Saharan Africa. Journal of lower genital tract disease.

[8] Vandepitte J, Bukenya J, Weiss HA, Nakubulwa S, Francis SC, Hughes P (2011). HIV and other sexually transmitted infections in a cohort of women involved in high risk sexual behaviour in Kampala, Uganda. Sexually transmitted diseases.

[9] De Marco F, Houissa-Kchouk F, Khelifa R, Marcante ML (2006). High‐risk HPV types in Tunisia. A pilot study reveals an unexpectedly high prevalence of types 58 and 82 and lack of HPV 18 among female prostitutes. Journal of medical virology.

[10] Veldhuijzen NJ, Braunstein SL, Vyankandondera J, Ingabire C, Ntirushwa J, Kestelyn E (2011). The epidemiology of human papillomavirus infection in HIV-positive and HIV-negative high-risk women in Kigali, Rwanda. BMC Infect Dis.

[11] Wanyenze RK, Musinguzi G, Kiguli J, Nuwaha F, Mujisha G, Musinguzi J (2017). When they know that you are a sex worker, you will be the last person to be treated”: perceptions and experiences of female sex workers in accessing HIV services in Uganda. BMC international health human rights.

[12] Arbyn M, Sankaranarayanan R, Muwonge R, Keita N, Dolo A, Mbalawa CG (2008). Pooled analysis of the accuracy of five cervical cancer screening tests assessed in eleven studies in Africa and India. International journal of cancer.

[13] Fokom-Domgue J, Combescure C, Fokom-Defo V, Tebeu PM, Vassilakos P, Kengne AP, et al. Performance of alternative strategies for primary cervical cancer screening in sub-Saharan Africa: systematic review and meta-analysis of diagnostic test accuracy studies. Bmj. 2015;351. 10.1136/bmj.h3084 PMC4490835 26142020

[14] Parashari A, Singh V (2013). Reasons for variation in sensitivity and specificity of visual inspection with acetic acid (VIA) for the detection of pre-cancer and cancer lesions of uterine cervix. Asian Pac J Cancer Prev.

[15] Onyenwenyi AO, Mchunu GG. Primary health care workers’ understanding and skills related to cervical cancer prevention in Sango PHC centre in south-western Nigeria: a qualitative study. Prim Health Care Res Dev. 2019;20. 10.1017/S1463423619000215 PMC6609971 32799996

[16] Sanghvi H, Limpaphayom KK, Plotkin M, Charurat E, Kleine A, Lu E (2008). Cervical cancer screening using visual inspection with acetic acid: operational experiences from Ghana and Thailand. Reprod Health Matters.

[17] Health. Mo. Strategic Plan for Cervical Cancer Prevention and Control in Uganda –2014 2010..

[18] Afzal O, Lieber M, Dottino P, Beddoe AM (2017). Cervical cancer screening in rural South Africa among HIV-infected migrant farm workers and sex workers. Gynecologic oncology reports.

[19] Luchters SM, Broeck DV, Chersich MF, Nel A, Delva W, Mandaliya K (2010). Association of HIV infection with distribution and viral load of HPV types in Kenya: a survey with 820 female sex workers. BMC Infect Dis.

[20] Organization WH. WHO guidelines for screening and treatment of precancerous lesions for cervical cancer prevention: World Health Organization; 2013. 24716265

[21] Organization WH. Prevention of cervical cancer through screening using visual inspection with acetic acid (VIA) and treatment with cryotherapy. A demonstration project in six African countries: Malawi, Madagascar, Nigeria, Uganda, the United Republic of Tanzania, and Zambia. World Health Organization. 2012.

[22] Health WHOR, Organization WH, Diseases WHOC, Promotion H. Comprehensive cervical cancer control: a guide to essential practice: World Health Organization; 2006. 25642554

[23] Heatley M (2002). How should we grade CIN?. Histopathology.

[24] Health UMo. Uganda clinical guidelines 2016. 2016.

[25] Ministry of Health (2016). Consolidated guidelines for prevention and treatment of HIV in Uganda.

[26] Babor TF, de la Fuente JR, Saunders J, Grant M. The Alcohol Use Disorders Identification Test: Guidelines for use in. 2001.

[27] Ajenifuja KO, Gage JC, Adepiti AC, Wentzensen N, Eklund C, Reilly M, et al. A population-based study of visual inspection with acetic acid (VIA) for cervical screening in rural Nigeria. Int J Gynecologic Cancer. 2013;23(3). 10.1097/IGC.0b013e318280f395 PMC3580031 23354369

[28] Pfaff C (2018). Early experiences in integrating cervical cancer screening and treatment into HIV services in Zomba Central Hospital, Malawi. Malawi Medical Journal.

[29] Joshi S, Kulkarni V, Darak T, Mahajan U, Srivastava Y, Gupta S (2015). Cervical cancer screening and treatment of cervical intraepithelial neoplasia in female sex workers using “screen and treat” approach. International Journal of Women’s Health.

[30] Denny L, Prendiville W (2015). Cancer of the cervix: Early detection and cost-effective solutions. International Journal of Gynecology Obstetrics.

[31] Horo A, Jaquet A, Ekouevi DK, Toure B, Coffie PA, Effi B (2012). Cervical cancer screening by visual inspection in Cote d’Ivoire, operational and clinical aspects according to HIV status. BMC Public Health.

[32] Moon TD, Silva-Matos C, Cordoso A, Baptista AJ, Sidat M, Vermund SH (2012). Implementation of cervical cancer screening using visual inspection with acetic acid in rural Mozambique: successes and challenges using HIV care and treatment programme investments in Zambézia Province. J Int AIDS Soc.

[33] Poli UR, Bidinger P, Gowrishankar S (2015). Visual inspection with acetic acid (via) screening program: 7 years experience in early detection of cervical cancer and pre-cancers in rural South India. Indian journal of community medicine: official publication of Indian Association of Preventive Social Medicine.

[34] Soohoo M, Blas M, Byraiah G, Carcamo C, Brown B (2013). Cervical HPV infection in female sex workers: a global perspective. The open AIDS journal.

[35] Couture M-C, Page K, Stein ES, Sansothy N, Sichan K, Kaldor J (2012). Cervical human papillomavirus infection among young women engaged in sex work in Phnom Penh, Cambodia: prevalence, genotypes, risk factors and association with HIV infection. BMC Infect Dis.

[36] Ministry of Health (2017). Uganda Population-Based HIV Impact Assessment (UPHIA) 2016–2017.

[37] Mutyaba T, Mirembe F, Sandin S, Weiderpass E (2010). Evaluation of’see-see and treat’strategy and role of HIV on cervical cancer prevention in Uganda. Reproductive health.

[38] Nyanzi S, Nyanzi B, Kalina B, Pool R (2004). Mobility, sexual networks and exchange among bodabodamen in southwest Uganda. Culture Health Sexuality.

[39] Mbonye M, Nakamanya S, Nalukenge W, King R, Vandepitte J, Seeley J (2013). ‘It is like a tomato stall where someone can pick what he likes’: structure and practices of female sex work in Kampala, Uganda. BMC Public Health.

